# Prolonged Use of Proton Pump Inhibitors, but Not Histamine-2 Receptor Antagonists, Is Associated With Lower Bone Mineral Density in Males Aged Over 70

**DOI:** 10.3389/fmed.2021.725359

**Published:** 2021-08-23

**Authors:** Shihua Gao, Wenting Song, Tianye Lin, Wenhuan Chen, Wei He, Qiushi Wei, Ziqi Li

**Affiliations:** ^1^Guangzhou University of Chinese Medicine, Guangzhou, China; ^2^Department of Orthopaedics, The Third Affiliated Hospital, Guangzhou University of Chinese Medicine, Guangzhou, China

**Keywords:** proton pump inhibitor (PPI), histamine-2 receptor antagonist (H2RA), bone mineral density (BMD), elderly, national health and nutrition examination survey (NHANES)

## Abstract

**Aims:** The association of acid suppressants use with bone mineral density (BMD) is still unclear, especially in older adult with prolonged use of proton pump inhibitors (PPIs) or histamine-2 receptor antagonists (H2RAs). In this study, our aim was to investigate the association between PPI or H2RA use and BMD in general US older adults.

**Methods:** We conducted a cross-sectional study on a sample from National Health and Nutrition Examination Survey. Association between long-term use of PPIs or H2RAs and lumber spine BMD in elderly was evaluated using weighted multivariate linear regression models. Sensitive and subgroup analysis were also performed in this study.

**Results:** Long-term PPI use is correlated with lower lumber spine BMD in our multivariable regression model after adjusting for known confounding factors. Further analysis showed PPI use with a duration over 1 year was negatively associated with lumber spine BMD in male, elderly aged over 70 years, and white elderly. There is no significant association between long-term H2RA use and lumber spine BMD.

**Conclusions:** Our results indicated that the association between long-term use of PPI and lumber spine BMD differed by gender. Long term use of PPIs would reduce lumber spine BMD in older men, while H2RA use is not significantly linked with lumber spine BMD. Patients that are at high risk of bone loss should shortened the duration of PPI use (<1 year) or use H2RAs as alternative if possible.

## Introduction

Bone metabolism is responsive to multiple stimuli, including hormonal change, aging, and immunologic inputs (1, 2). Because of the responsiveness of skeleton system, many commonly used medications suspected to have deleterious effect on bone health. Previous work has suggested that acid suppressants may have negative impact on bone repair and remodeling by interfering with the function of osteoclasts (3). Acid suppressants such as proton pump inhibitors (PPIs) and histamine-2 receptor antagonists (H2RAs) are among the most frequently prescribed medications around the world (4). Some patients may require prolonged use of PPIs or H2RAs because of recurrence of the diseases like gastroesophageal reflux disease and peptic ulcers, or to prevent peptic ulcers from long-term used of medications like non-steroidal anti-inflammatory drugs (NSAIDs) or aspirin (5).

There are several studies examining the effect of acid suppressants on bone health, but these findings have been conflicting. Multiple studies indicated the use of PPIs or H2RAs is associated with higher risk of fracture or bone loss (6–9). However, result from a PPI user cohort indicated there is no association between long-term use of PPI and bone mineral density (BMD) (10). A recent study comparing two categories of acid suppressants indicated PPI users have a higher risk of osteoporotic fracture compared to H2RA users (11). In a study focusing on patients receiving kidney transplantation, result showed only PPI use is linked with osteoporosis but not H2RA (12). The effects of acid suppressants on bone health are still inconsistent based on different study population.

Currently, evidence linking long-term PPI or H2RA use with BMD in older general population is limited. In addition, BMD could be affected by multiple factors, including demographic difference, nutrition status, and lipid metabolism (13, 14). These factors should be included in the analysis as confounders to establish a more accurate conclusion. Therefore, we performed a cross-sectional study based on the national population to investigate the association of long-term use of PPI and H2RA with BMD in older adults.

## Materials and Methods

### Study Population

The National Health and Nutritional Examination Survey (NHANES) is a national survey of the American population with a complex, stratified, multistage, probability sampling method. Details about this cross-sectional survey are available at www.cdc.gov/nchs/nhanes. The data of this study were from four circles of National Health and Nutritional Examination Survey (NHANES), from 1999 to 2006.

The study population was limited to older adults aged over 60 with complete data on acid suppressant use (including PPIs and H2RAs), BMD, and covariate. Participants were excluded if they were using two types of acid suppressants simultaneously or the duration of acid suppressants use was <90 days.

**Figure 1:**
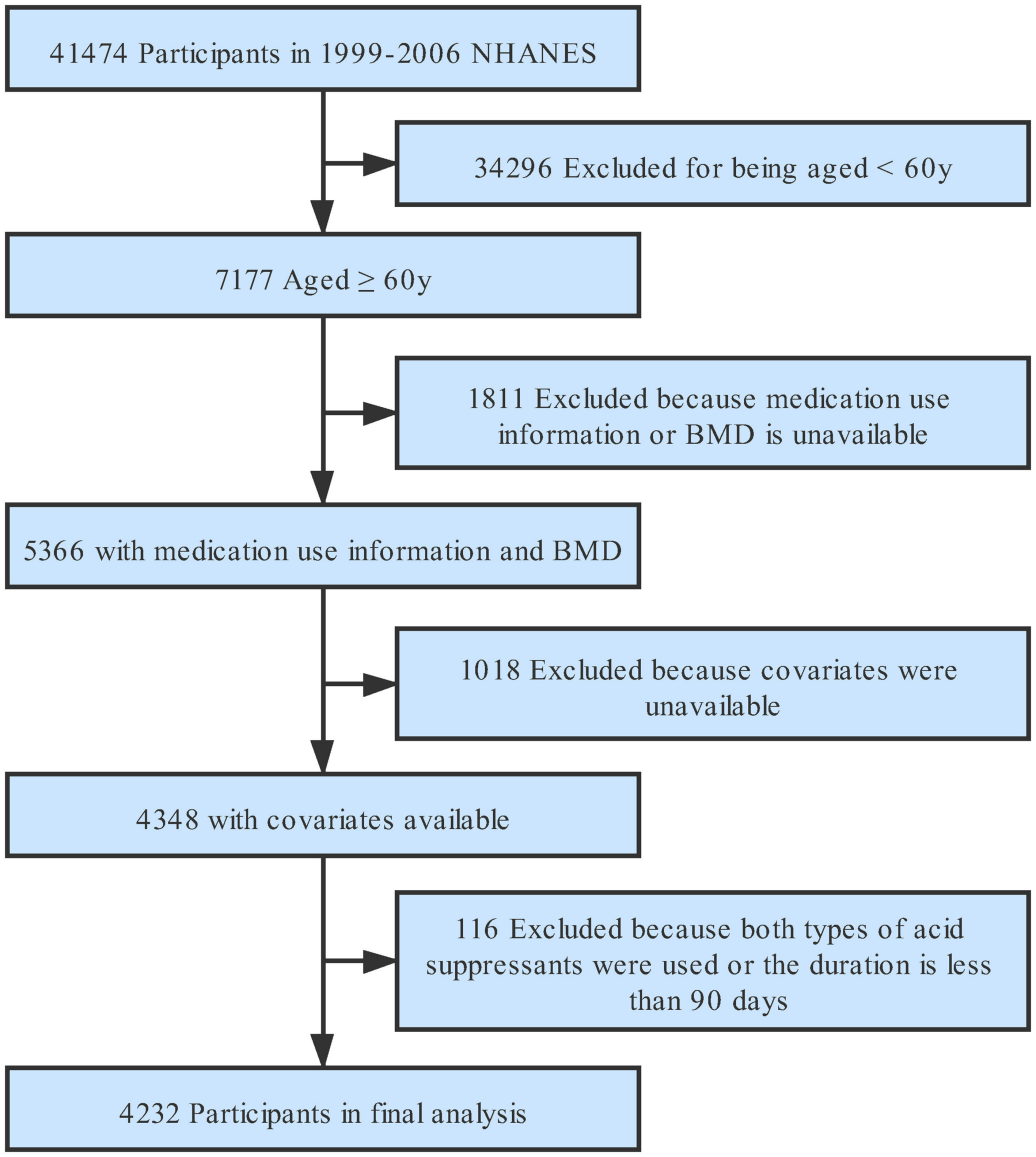
Study flowchart. Flow chart showing the process of participants selection. Of 41,474 participants from 1999 to 2005 National Health and Nutrition Examination Survey (NHANES), 4,232 remained in the final analysis. BMD, bone mineral density.

Of the 41,474 participants in 1999 to 2006 NHANES, there were 7,177 aged 60 years and older; 1,811 were missing data on prescription medication use information or BMD; another 1,018 were missing data on covariate; 116 of these older adults were taking two types of acid suppressant at the same time or the duration of medication use was <90 days, leaving 4,232 participants in the final analysis (Figure 1).

### Variables

The independent variables in this study were long-term PPI and H2RA uses. The types and duration of acid suppressants use were acquired from prescription medications questionnaires. The types of PPI included omeprazole, esomeprazole, lansoprazole, pantoprazole, and rabeprazole. The types of H2RA taken by included participants were cimetidine, ranitidine, famotidine, and nizatidine. Dependent variables were lumbar spine BMD measured by dual-energy X-ray absorptiometry.

The following covariates were included in the analysis: age, race, education, physical activity, body mass index, cholesterol, triglycerides, total protein, serum albumin, alkaline phosphatase, serum calcium, serum phosphorus, total calcium intake (including calcium from diet and supplements), and use of other medications (including oral or inhaled corticosteroids, bisphosphate, NSAIDs, or aspirin). The detailed acquisition process and measuring method of each variable are available at www.cdc.gov/nchs/nhanes.

### Statistical Analysis

We calculated estimates accounting for NHANES sampling weights. Data were reported as mean ± standard deviation for continuous variables and as percentages or frequency for categorical variables. Weighted linear regression model was used for continuous variables and weighted χ2 test for categorical variables to estimate the difference among groups. Weighted multiple regression analyses were adopted to evaluate the independent relationship between acid suppressants use and lumber spine BMD. We constructed three distinct models using weighted univariate and multivariate linear regression model, including non-adjusted model (no covariate was adjusted), minimally adjusted model (only demographic variables were adjusted), and fully adjusted model (covariates presented in Table 1 were adjusted). Effect sizes with 95% confidence intervals were recorded. *P-*values <0.05 (two-sided) were considered statistically significant. Modeling was performed with the statistical software packages R (http://www.R-project.org, The R Foundation).

**Table 1:** Weighted characteristics of 4,232 participants included in this study.

**Characteristics**	**Non-user (** ***n*** **= 3682)**	**PPI user (** ***n*** **= 381)**	**H2RA user (** ***n*** **= 169)**	***P*** **-value**
*Age (%)*				
<70 years	57.32	58.98	51.43	0.2001
≥70 years	42.68	41.02	48.57	
Men (%)	43.09	43.44	43.20	0.9906
*Race/ethnicity (%)*				
White	81.05	85.07	88.03	0.1298
Black	8.08	6.95	4.84	
Mexican-American	3.63	2.14	2.39	
Other Hispanic	3.60	1.91	2.07	
Other	3.64	3.93	2.67	
*Education (%)*				
Lower than high school	27.43	26.48	28.12	0.8724
High school	29.10	31.55	29.94	
More than high school	43.47	41.96	41.94	
*Physical activity (%)*				
Sedentary	25.78	28.62	33.05	0.0015
Low	27.58	23.22	24.43	
Moderate	17.60	23.63	12.58	
High	29.03	24.52	29.94	
Body mass index (kg/m2)	28.42 ± 5.69	29.23 ± 5.91	29.70 ± 6.13	0.0004
Total cholesterol (mg/dl)	209.64 ± 41.13	203.23 ± 42.23	207.69 ± 38.72	0.0096
Triglycerides (mg/dl)	153.17 ± 107.48	153.24 ± 76.26	180.08 ± 113.10	0.0023
Total protein (g/dl)	7.24 ± 0.49	7.12 ± 0.51	7.23 ± 0.59	<0.0001
Serum albumin (g/dl)	4.23 ± 0.31	4.17 ± 0.32	4.24 ± 0.32	0.0007
Alkaline phosphatase (U/L)	76.15 ± 29.76	77.33 ± 29.48	76.29 ± 25.79	0.7403
Serum calcium (mg/dl)	9.50 ± 0.41	9.52 ± 0.43	9.51 ± 0.39	0.7843
Serum phosphorus (mg/dl)	3.63 ± 0.54	3.71 ± 0.56	3.67 ± 0.69	0.0059
Total calcium intake (mg/d)	1058.77 ± 764.61	1029.76 ± 710.33	1110.32 ± 823.95	0.4733
Oral or inhaled corticosteroids (%)	5.31	11.31	16.71	<0.0001
Bisphosphate (%)	1.66	0.75	0.41	0.1570
NSAIDs or aspirin (%)	13.25	20.84	21.64	<0.0001
*Duration of acid suppressant (%)*				
≤ 1 year	0	33.95	18.82	<0.0001
>1 year	0	66.05	81.18	
Lumber spine BMD (g/cm^2^)	1.01 ± 0.18	1.00 ± 0.17	1.04 ± 0.19	0.0429

*Mean ± SD for continuous variables and P-value was calculated by weighted linear regression model. % for categorical variables and P-value was calculated by weighted χ2 test*.

## Result

A total of 4,232 participants aging over 60 were included in this study. Participants were classified into non-user, PPI user, and H2RA user. The weighted characteristics of these participants were shown in Table 1. About 43% were male, and over 80% were white in each group. Among these participants, the three exposure groups were similar in age, gender, race, education level, alkaline phosphatase, serum calcium, total calcium intake, and bisphosphate, while physical activity level, body mass index, total cholesterol, triglycerides, total protein, serum albumin, serum phosphorus, corticosteroids use, NSAIDs or aspirin use, duration of acid suppressant use, and lumber spine BMD were all significantly different. On the other hand, the distribution of duration of acid suppressive therapy was similar between different age groups (Table 2).

**Table 2:** Weighted distribution of duration of acid suppressant use in different age groups.

	** <70 years**	**≥70 years**	***P*** **-value**
*Duration of PPI use (%)*			0.7644
≤ 1 year	33.35	34.82	
>1 year	66.65	65.18	
*Duration of H2RA use (%)*			0.5737
≤1 year	17.18	20.56	
>1 year	82.82	79.44	

*P-value was calculated by weighted χ2 test*.

**Figure 2:**
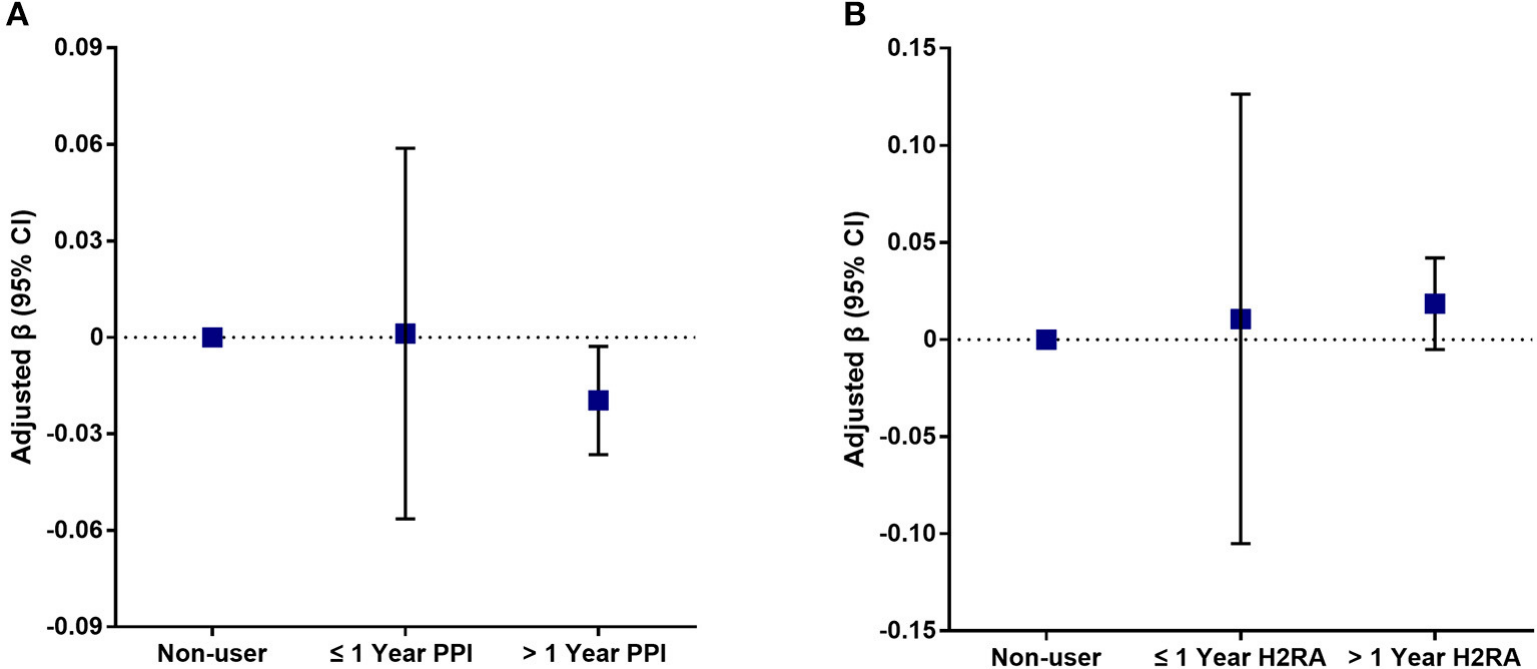
Association of different durations of acid suppressant use with lumber spine BMD. Plot **(A)** shows the association between different durations of proton pump inhibitor use and lumber spine BMD. Plot **(B)** shows the association between different durations of histamine-2 receptor antagonist use and lumber spine BMD. Age, gender, race/ethnicity, education, physical activity, body mass index, total cholesterol, triglycerides, total protein, serum albumin, alkaline phosphatase, serum calcium, serum phosphorus, total calcium intake, corticosteroids use, bisphosphate use, NSAIDs, or aspirin use was adjusted. Non-user was defined as reference.

Weighted univariate and multivariate linear regression models were constructed (Table 3), and two types of acid suppressants were compared to non-user separately. No significant association between PPI use and lumber spine BMD was found in the unadjusted model (model 1). However, in the fully adjusted model (model 3), there were significant association between PPI use and lumbar spine BMD [−0.0181 (−0.0344, −0.0018)]. Sensitive analysis showed a longer duration of PPI use (>1 year) had similar negative association [−0.0196 (−0.0364, −0.0028)] with lumber spine BMD, and linear relationship was also shown between duration of PPI use and lumber spine BMD (*P* for trend 0.0242). On the other hand, there was a positive association between long-term H2RA use and lumber spine BMD. However, the estimate was not significant in the fully adjusted model and sensitive analysis showed similar results. The results of sensitive analysis were also demonstrated in Figure 2.

**Table 3:** Association of long-term acid suppressant use with lumber spine bone mineral density in 4,232 participants aged over 60 years.

**Exposure**	**PPI**			**H2RA**		
	**Model 1**	**Model 2**	**Model 3**	**Model 1**	**Model 2**	**Model 3**
*Long-term acid suppressant use*						
β (95%CI)	−0.0097 (−0.0279, 0.0086)	−0.0106 (−0.0276, 0.0064)	−0.0181 (−0.0344, −0.0018)	0.0312 (0.0052, 0.0572)	0.0324 (0.0082, 0.0567)	0.0182 (−0.0050, 0.0413)
*P*-value	0.2984	0.2210	0.0293	0.0189	0.0089	0.1237
***Sensitive analysis***						
*Duration ≤ 1 year*						
β (95%CI)	−0.0249 (−0.0902, 0.0405)	−0.0169 (−0.0778, 0.0440)	0.0012 (−0.0564, 0.0588)	−0.0079 (−0.1395, 0.1237)	−0.0008 (−0.1235, 0.1219)	0.0106 (−0.1051, 0.1264)
*P*-value	0.4559	0.5863	0.9665	0.9064	0.9895	0.8572
*Duration > 1 year*						
β (95%CI)	−0.0085 (−0.0274, 0.0103)	−0.0101 (−0.0277, 0.0074)	−0.0196 (−0.0364, −0.0028)	0.0327 (0.0063, 0.0592)	0.0337 (0.0090, 0.0584)	0.0185 (−0.0051, 0.0421)
*P*-value	0.3757	0.2583	0.0225	0.0154	0.0075	0.1247
*P* for trend	0.3350	0.2369	0.0242	0.0164	0.0078	0.1224

*Model 1: no covariate was adjusted. Model 2: age, gender, and race/ethnicity were adjusted*.

*Model 3: age, gender, race/ethnicity, education, physical activity, body mass index, total cholesterol, triglycerides, total protein, serum albumin, alkaline phosphatase, serum calcium, serum phosphorus, total calcium intake, corticosteroids use, bisphosphate use, NSAIDs, or aspirin use was adjusted*.

*Reference category was non-user*.

**Figure 3:**
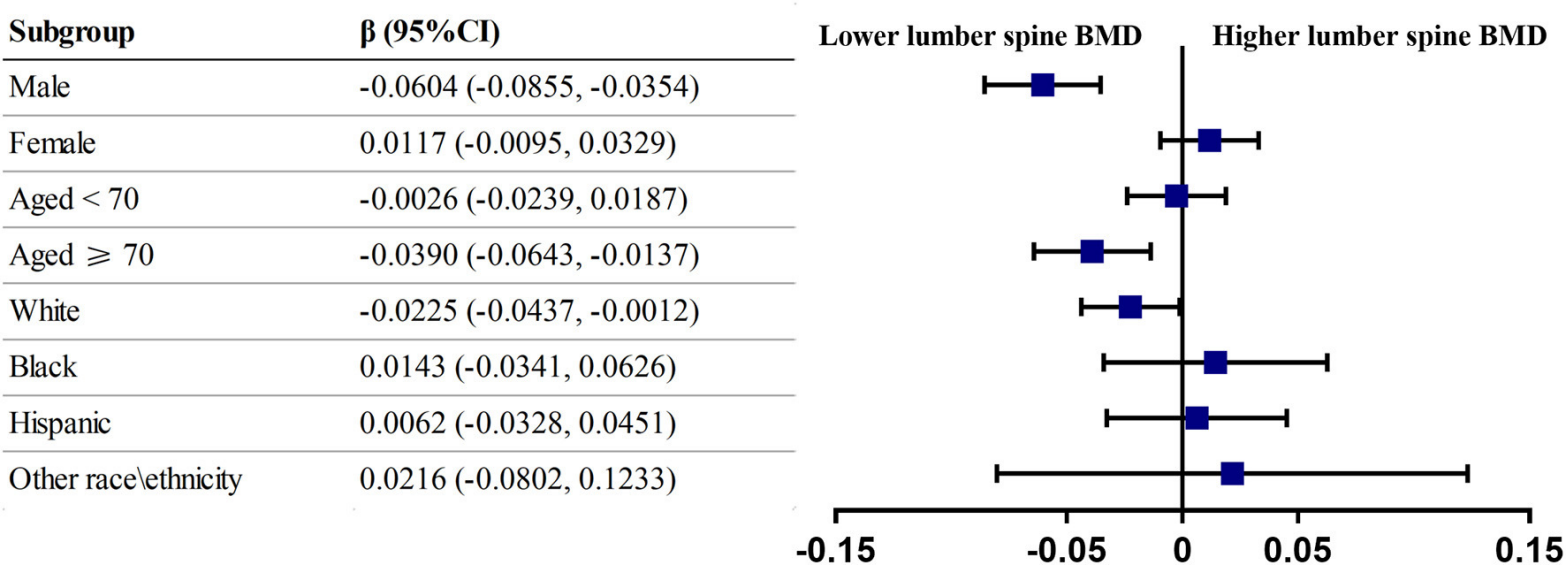
Subgroup analysis of long-term PPI use. Forest plot showing the association between PPI use and lumber spine BMD for various subgroups. Age, gender, race/ethnicity, education, physical activity, body mass index, total cholesterol, triglycerides, total protein, serum albumin, alkaline phosphatase, serum calcium, serum phosphorus, total calcium intake, corticosteroids use, bisphosphate use, NSAIDs, or aspirin use was adjusted except for the stratification variable itself. Reference category was non-user.

Analysis of the PPI-BMD association stratified by demographic variables (Figure 3) showed that the estimates remained statistically significant in male participants [−0.0604 (−0.0855, −0.0354)], participants aged over 70 years [−0.0390 (−0.0643, −0.0137)], and white participants [−0.0225 (−0.0437, −0.0012)].

## Discussion

This study was set out to investigate whether long-term use of PPIs or H2RAs is independently associated with lumber spine BMD. The studied population was a nationally representative and large sample of US older adults aged 60 years and over. Results from our study suggested that using PPIs for more than 1 year was negatively associated with lumber spine BMD in male, elderly aged over 70 years, and white elderly. An insignificant positive association between long-term H2RA use and lumber spine BMD was observed.

Prior studies examining bone loss and risk of osteoporotic fracture in people who use PPIs or H2RAs have shown mixed results, and the association between these potent acid suppressants and bone loss remained debatable. Most results from previous longitudinal studies suggested PPI use was not associated with lower BMD (15–17). However, a meta-analysis reported PPI use was related to osteoporotic fractures, indicating PPIs may induce bone loss (18). Also, there are cross-sectional studies demonstrating negative association between PPI use and BMD, which is similar to our current study (7, 19). On the contrary, no significant relationship was found between H2RAs and bone loss or fracture in previous studies as well as in our current study (12, 15, 17, 20). We followed the Strengthening the Reporting of Observational Studies in Epidemiology (STROBE) guideline to perform subgroup analyses to further investigate the relationship in different population groups (21). When stratified by gender, the association of PPI use with BMD was not significant in women. Interestingly, result from the Study of Women Across the Nation (SWAN) cohort focusing on perimenopausal women also failed to found evidence of bone loss in PPI users (17). Another study from the Study of Osteoporotic Fractures (SOF) cohort also yield similar result that PPI use was not associated with low BMD in older women (15). The mechanism under this gender difference is unclear and worth being further studied.

Unlike previous cross-sectional studies, we excluded participants taking PPIs or H2RAs for <90 days in the current study. Most large sample studies investigating the effect of acid suppressants on bone loss included all participants with any use of PPIs or H2RAs, which may induce potential bias because the cumulative dose of acid suppressants may be largely different (11, 15, 17). To our knowledge, only one study specifically aimed at the association between long-term exposure of PPIs and BMD. Result from this relatively smaller scale cross-sectional study indicated that PPI use of more than 5 years is not associated with significant change in BMD or bone strength (10). However, we discovered that the negative association between PPI use and BMD may have a potential does cumulative effect. Participants with extend duration of PPI use (>365 days) have significantly lower lumber spine BMD. Furthermore, we stratified the participants based roughly on the mean age of all participants (70 years). Subgroup analysis showed a significant association of long-term use of PPIs with lower BMD in participants aged over 70 years. We assumed the negative association between PPI uses with BMD becomes significant in an older age group because bone loss is more likely to happen in the process of aging.

One explanation of the deleterious effect of acid suppressants on BMD is that the anti-acid properties may potentially interfere with the absorption of calcium, which is believed to be crucial for bone health (22). Several studies reported short-term use of omeprazole decreased serum calcium and fractional absorption of calcium (23–25). Theoretically, PPIs have better anti-acid effect than H2Ras (26). Therefore, it can be explained that H2RAs have little interference on the absorption of nutrients like calcium and seldom induce bone loss. Of interest, we failed to observe a significant difference of serum calcium level and amount of daily calcium intake among non-user, PPI user, and H2RA user, but our result still showed an association of PPI use with lower BMD. A recent study demonstrated that pantoprazole accelerated bone turnover by affecting the number of osteoclasts and decreased the expression of bone morphogenetic protein-4 (BMP-4) in aged mice, indicating PPIs may affect bone metabolism by regulating bone formation (27).

In the current study, we used multiracial samples representative of US general population for better generalizability. In addition, the sample size of our study is large enough to perform subgroup analysis to better identified population groups that are more susceptible to PPI related bone loss. However, there are several limitations in this study. Firstly, because of the cross-sectional nature of the study design, the causal relation between PPI use and lower BMD is hard to be determined through our results. Secondly, there may be other confounding factors that can alter our observed results, which is also an inherent limitation of observational study design. For example, co-morbidities of the subjects were not included in our analysis because the type of co-morbidities that may potentially affects BMD was still unclear. Lastly, although NHANES questionnaires are sophisticatedly designed, the exact information of PPI use or H2RA use is hard to acquire because this survey only reports on prescription medication. Therefore, we were unable to capture all participants that were using PPIs or H2RAs because some of them may be taking these acid suppressants from over-the counter-source, increasing the potential risk of bias. Likewise, dosage and reasons of medication use were unavailable in the NHANES dataset. Longitudinal studies with long duration of follow-up and large sample size will be needed to further confirm the association of PPIs and H2RAs with bone loss.

In conclusion, this nationwide cross-sectional study indicated that long-term use is associated with lower lumber spine BMD in men or advanced age group (>70 years), while H2RA use is not significantly linked with lumber spine BMD. These findings may have clinical implication about the use of acid suppressants. In patients that are at high risk of bone loss, the duration of PPI use should be shortened (<1 year) or changed to H2RAs if possible. However, further basic and clinical studies are needed to confirm our findings.

## Data Availability Statement

Datasets of this study are all available at https://wwwn.cdc.gov/nchs/nhanes/.

## Ethics Statement

The studies involving human participants were reviewed and approved by National Center for Health Ethics Review Board. The patients/participants provided their written informed consent to participate in this study.

## Author Contributions

SG and WS: drafting of the manuscript. QW and TL: review and editing. ZL and WH: concept and design. WC: data collection.

## Conflict of Interest

The authors declare that the research was conducted in the absence of any commercial or financial relationships that could be construed as a potential conflict of interest.

## Publisher's Note

All claims expressed in this article are solely those of the authors and do not necessarily represent those of their affiliated organizations, or those of the publisher, the editors and the reviewers. Any product that may be evaluated in this article, or claim that may be made by its manufacturer, is not guaranteed or endorsed by the publisher.
